# The −930A>G polymorphism of the *CYBA* gene is associated with premature coronary artery disease. A case–control study and gene–risk factors interactions

**DOI:** 10.1007/s11033-014-3191-9

**Published:** 2014-01-31

**Authors:** Pawel Niemiec, Tomasz Nowak, Tomasz Iwanicki, Jolanta Krauze, Sylwia Gorczynska-Kosiorz, Wladyslaw Grzeszczak, Anna Ochalska-Tyka, Iwona Zak

**Affiliations:** 1 Department of Biochemistry and Medical Genetics, School of Health Sciences, Medical University of Silesia, Medykow Str 18, 40-752 Katowice, Poland; 2 1st Department of Cardiac Surgery in Upper Silesian Center of Cardiology in Katowice, School of Medicine, Medical University of Silesia, Ziolowa Str 47, 40-635 Katowice, Poland; 3 Department of Internal Medicine, Diabetes and Nephrology, School of Medicine, Medical University of Silesia, 3 maja Str 13-18, 41-800 Zabrze, Poland; 4 Regional Centre of Blood Donation and Blood Treatment in Raciborz, Sienkiewicza Str 3, 47-400 Racibórz, Poland

**Keywords:** CYBA, Polymorphism, NADPH oxidase, CAD, Atherosclerosis

## Abstract

Reactive oxygen species (ROS) are involved in the pathogenesis of atherosclerosis and coronary artery disease (CAD). NADPH oxidases are the main source of ROS in the vasculature. p22phox is a critical component of vascular NADPH oxidases and is encoded by the *CYBA* (cytochrome b_245_ alpha) gene. The −930A>G *CYBA* polymorphism (rs9932581:A>G) modulates the activity of the *CYBA* promoter, and influences *CYBA* transcriptional activity. The aim of the present study was to analyze a possible association between the −930A>G polymorphism and CAD and to search for gene–traditional risk factors interactions. 480 subjects were studied: 240 patients with premature CAD, 240 age and sex matched blood donors. The −930A>G polymorphism was genotyped using the TaqMan^®^ Pre-designed SNP Genotyping Assay (Applied Biosystems). The −930G allele carrier state was a risk factor for CAD (OR 2.03, 95 % CI 1.21–3.44, *P* = 0.007). A synergistic effect of the −930G allele with overweight/obesity (BMI ≥ 25) and cigarette smoking was found. The estimated CAD risk for BMI ≥ 25 and the −930G allele interaction was about 160 % greater than that predicted by assuming additivity of the effects, and about 40 % greater for interaction of cigarette smoking and the −930G allele. Overweight/obesity was a risk factor for CAD only in the −930G allele carriers (*P* < 10^−10^) but not in the AA homozygotes (*P* = 1.00). In conclusion the −930A>G *CYBA* polymorphism is associated with CAD in the Polish population. The −930G allele carriers are particularly at risk of consequences of obesity and tobacco smoke exposure.

## Introduction

Reactive oxygen species (ROS) are involved in the pathogenesis of atherosclerosis almost at every stage. Their participation is already noticeable at the stage of promotion of the endothelial dysfunction and is often evident during the development and destabilization of the atherosclerotic plaque [1].

NADPH oxidases are considered as the main source of superoxide anion (O_2_^−^) in the cardiovascular system. They are multicomponent enzymes composed of the membrane-bound cytochrome b_558_ (catalytic NOX protein and p22phox) and many auxiliary cytoplasmic subunits for different types of NOX proteins. NOX proteins differ in their tissue distribution and regulation of the activity and expression [1, 2]. The expression of NOX1, NOX2, NOX4 and NOX5 was demonstrated in endothelial cells as well as vascular smooth muscle cells [1]. The most important role in the vascular production of O_2_^−^ is assigned to NADPH oxidases containing the NOX2 protein, which is most strongly expressed in phagocytic cells [1]. p22phox is a common and constant element of NADPH oxidases complexes containing NOX1–NOX4 proteins, and plays a key role in the cytochrome b_558_ stabilization and is necessary to the initiation of O_2_^−^ production by NOX1–NOX4 proteins [1].

p22phox is encoded by the *CYBA* (light chain of cytochrome b_558_, cytochrome b_245_ alpha) gene (16q24). A significant number of genetic polymorphisms has been reported both in the exons and non-coding sequences, especially in the promoter region of *CYBA* [2, 3]. For some of them an impact on the *CYBA* expression and the superoxide production by NADPH oxidases was suggested.

The −930A>G polymorphism (rs9932581:A>G) is one of the promoter polymorphisms, located at the nucleotide position −930 from the ATG codon [4]. This polymorphism is situated in the potential binding site of C/EBP (*CCAAT/enhancer*-*binding protein*) transcription factors. It was speculated that the −930A>G polymorphism modulates the activity of the *CYBA* promoter, and therefore transcriptional activity of *CYBA*, through the differential allele-dependent binding of C/EBP [5]. The −930G allele was the one that increased the affinity of C/EBP to the promoter [5]. A functional analysis revealed that the −930G allelic *CYBA* promoter has a 30 % higher (*P* < 0.05) gene expression than the −930A allelic promoter [4]. However, the studies on the functional impact of the −930A>G polymorphism on the NADPH oxidase-dependent superoxide production often revealed conflicting results. In some studies, especially those conducted in hypertensive individuals [5, 6], the −930G allele was related to an increased O_2_^−^ production. In other cases, it did not affect the superoxide synthesis at all [7–9]. These discrepancies may result from intra- and inter-individual variability in the NADPH oxidase activity, different methods of O_2_^−^ measurements and the use of different cell lines in the experiments.

Association studies of the −930A>G polymorphism with cardiovascular diseases are numerous. To date, the −930G allele was considered as a genetic marker associated with hypertension in the Spanish population [4, 6] and Japanese males [10]. In the present work, we tried to analyze a possible association between the −930A>G polymorphism and coronary artery disease and to analyze potential interactions of *CYBA* alleles and traditional risk factors of atherosclerosis and CAD.

## Materials and methods

### Participants

We studied 480 subjects. Group 1: 240 patients with angiographically proven premature CAD, 72 women and 168 men, aged 27–55 years (mean 44.55 ± 6.09). Group 2: 240 blood donors (BD) including 70 women and 170 men, aged 27–55 years (mean 43.88 ± 8.21). CAD subjects were selected from (1) patients admitted to the 1st Department and Clinic of Cardiology at the Upper Silesian Centre of Cardiology in Katowice; (2) patients admitted to the 1st Department of Cardiac Surgery at the Upper Silesian Centre of Cardiology in Katowice. Patients were classified for the study by the same cardiologist. Controls were recruited from the Regional Centre of Blood Donation and Blood Treatment in Katowice and the Regional Centre of Blood Donation and Blood Treatment in Raciborz. Following nationwide recommendations of the Polish Centres of Blood Donation and Blood Treatment, blood samples were obtained only from subjects with systolic blood pressure (BP) <140 and diastolic BP <90 on the day of blood collection. All subjects were Polish Caucasians, inhabitants of Upper Silesia.

Inclusion and exclusion criteria, details of the medical interview, diagnosis and evaluation as well as criteria for CAD, MI and traditional risk factors were described previously [11].

The study protocol was approved by the Ethics Committee of the Medical University of Silesia in Katowice (Poland) and all subjects gave written informed consents.

### Biochemical analyses

Total serum cholesterol (TC), HDL-cholesterol (HDL-chol) and triacylglycerols (TG) were measured by enzymatic methods (commercial Analco kit, Warsaw, PL). LDL-cholesterol (LDL-chol) levels were calculated according to the Friedewald formula [12] in subjects with TG levels below 4.4 mmol/l.

### Genetic analyses

Genomic DNA was extracted from peripheral lymphocytes using the MasterPure genomic DNA purification kit (Epicentre Technologies, Madison, USA). The −930A>G polymorphism of the *CYBA* gene was genotyped using the TaqMan^®^ Pre-designed SNP Genotyping Assay (Applied Biosystems, Foster City, California, USA). The total volume of 20 μl of reaction mix included: 10 μl of TaqMan^®^ Genotyping Master Mix (Cat.# 4371355), 1 μl of probe (TaqMan^®^ Pre-designed SNP Genotyping Assay, Cat.# 4351376, C_11291925_10), 1 μl of DNA template (15 ng/μl) and 8 μl of deionized water. The probe was diluted with the TE buffer (1:1) before the reaction. The polymerase chain reaction amplification was performed according to the manufacturer’s specifications. Genotyping was performed using the 7300 real-time PCR system (Applied Biosystems).

### Statistical analyses

Data were analyzed using *Statistica* *10.0* (STATSOFT, Tulusa, OK, USA) and *SAS 9.1* (SAS Institute Inc., NC, USA) software. Normality of distribution was checked by Shapiro–Wilk test and then a comparison of quantitative data was performed by Mann–Whitney *U* test (for variables with non-normal distribution) or the student’s *t* test (for variables with normal distribution). Allele frequencies were deduced from the genotype distribution. Hardy–Weinberg equilibrium was tested in all groups by a *χ* ^2^ test. Comparisons of genotypes and alleles frequencies between cases and control subjects were performed by a *χ* ^2^ test. When the number of subjects in the sample was lower than ten the Fisher’s correction was used. Statistical significance was accepted at *P* < 0.05. Odds ratios (OR) as well as their 95 % confidence intervals (CI) were computed using an univariate analysis (2 × 2 tables) and a multiple logistic regression analysis after adjustment for age, sex and traditional risk factors of coronary artery disease. Risk ratio values (95 % CI) were used when the number of individuals in any of the analyzed subgroups was 0. The effective sample size and statistical power of association analyzes were computed using *Epi Info™ 7.1.1.0* developed by Centers for Disease Control and Prevention (CDC).

Pearson’s correlation coefficients between *CYBA* variants and clinical and biochemical parameters were calculated. To determine possible synergistic/antagonistic interactions between *CYBA* genotypes and traditional risk factors of CAD, the 4 × 2 table approach of biological interactions was used. The synergy measures in an additive model were used to interpret the amount of interaction [13]. The interaction of the −930G allele with the respective factor was analyzed and AA homozygous subjects, not exposed to any specific risk factor, were used as a reference group (00 code). They were compared with subgroups of subjects exposed to only one of the factors (01—only traditional, 10—only genetic) and with a subgroup exposed to both factors (11 code).

Odds ratio values, obtained from 4 × 2 table comparisons, were used for a calculation of the synergy index (SI). The SI is a ratio of the observed effect with the joint exposure to genetic and traditional factors (OR_11_) divided by the effect predicted for the joint exposure assuming additivity of the effects observed in the presence of either a traditional or genetic factor (OR_01_ and OR_10_). No interaction corresponds to SI = 1, whereas SI > 1 can be interpreted as a measure of relative increase and SI < 1 of decrease in the effect among those exposed to both factors. The following formula of SI was used [13]:

$${\text{SI }} = {\text{ OR}}_{11} {-} \, 1/\left( {{\text{OR}}_{01} {-} \, 1} \right) \, + \, \left( {{\text{OR}}_{10} {-} \, 1} \right),$$

95 % CI for SIes were calculated using the SAS program described by Lundberg et al. [14].

An analysis of additive effects of traditional risk factors and *CYBA* genotypes was also performed in a standard univariate and multivariate logistic regression model. These were used to compare the frequency of carriers of the −930G allele or AA homozygotes exposed to traditional risk factor between CAD and blood donors groups.

## Results

Clinical and biochemical parameters of patients and controls are shown in Table 1. There were 72.1 % cases who had suffered from MI (*n* = 173) and 60.4 % patients with critical stenoses (>90 %) in coronary vessels (*n* = 145). CAD patients showed an increased level of TC, LDL cholesterol and TG, and a higher BMI value. The level of HDL cholesterol was significantly lower in CAD patients. The high value of the OR for hypertension (Table 1) resulted from the fact that, according to the nationwide recommendations of Polish Centres of Blood Donation and Blood Treatment, the blood samples were obtained only from blood donors with systolic BP less than 140 and diastolic BP less than 90 mmHg on the day of blood collection.

**Table 1:** Clinical and biochemical characteristics in the groups of coronary artery disease patients (CAD) and blood donors (BD)

Characteristic	CAD *n* = 240	BD *n* = 240	Crude OR (95 % CI) univariate analysis	*P*
Age (years), mean ± SD	44.55 ± 6.09	43.88 ± 6.22	–	0.22
Male gender, % (no.)	70.0 (168)	70.8 (170)	0.96 (0.65–1.42)	0.84
BMI, mean ± SD	27.15 ± 4.21	25.54 ± 3.36	–	<10^−3^
BMI ≥ 25, % (no.)	60.8 (146)	27.9 (67)	4.01 (2.73–5.88)	<10^−10^
Cigarette smoking, % (no.)	56.7 (136)	28.3 (68)	3.31 (2.26–4.83)	<10^−10^
Hypertension, % (no.)	57.1 (137)	2.3 (7)	44.27 (20.01–97.95)	<10^−10^
Diabetes mellitus, % (no.)	9.2 (22)	0 (0)	2.10 (1.91–2.31)^a^	<10^−7^
Familial history of CAD, % (no.)	34.2 (82)	0 (0)	2.52 (2.23–2.84)^a^	<10^−10^
TC (mmol/l), mean ± SD	5.76 ± 1.36	5.08 ± 1.21	–	<10^−7^
LDL (mmol/l), mean ± SD	3.98 ± 1.21	3.14 ± 1.17	–	<10^−10^
HDL (mmol/l), mean ± SD	1.12 ± 0.38	1.44 ± 0.57	–	<10^−10^
TG (mmol/l), mean ± SD	1.86 ± 0.98	1.40 ± 0.73	–	<10^−8^

^a^Risk ratio values (95 % CI), univariate analysis

### Analysis of the −930A>G polymorphism

Genotype frequencies were compatible with the Hardy–Weinberg equilibrium in both groups. Data from genotyping of the −930A>G polymorphism are shown in Table 2. Frequencies of the −930G allele carriers (GG+AG genotypes) were significantly higher in patients than in controls (*P* = 0.007). Results of logistic regression analysis confirmed that the −930G allele carrier state was a risk factor for CAD in the analyzed population (OR 2.03). The power of test was 77 %, with a 95 % two-sided confidence level. The −930A>G polymorphism was associated with CAD also after adjustment for traditional risk factors like sex, age, TC, LDL-chol, HDL-chol, TG, BMI (qualitative variable), diabetes mellitus, cigarette smoking status, hypertension and familial history of CAD. The results of multivariate analysis were: for the −930G allele carrier state: OR 2.22, 95 % CI 1.17–4.20, *P* = 0.015, for the AA genotype: OR 0.45, 95 % CI 0.24–0.86, *P* = 0.015.

**Table 2:** The frequency of genotypes and alleles of the −930A>G polymorphism of the *CYBA* gene in the groups of patients (CAD) and blood donors (BD)

Genotype, Allele	CAD (*n* = 240) % (n)	BD (*n* = 240) % (n)		OR (95 % CI), *P* univariate analysis
AA	10.4 (25)	19.2 (46)	Versus AG+GG	0.49 (0.29–0.82), 0.007
AG	51.7 (124)	47.1 (113)		–
GG	37.9 (91)	33.7 (81)	Versus AA+AG	*NS*
AA+AG	62.1 (149)	66.3 (159)	Versus GG	*NS*
GG+AG	89.6 (215)	80.8 (194)	Versus AA	2.03 (1.21–3.44), 0.007
−930A	36.2 (174)	42.7 (205)	Versus −930G	*NS*
−930G	63.8 (306)	57.3 (275)	Versus −930A	*NS*

*CAD* coronary artery disease patients, *BD* blood donors, *NS* no significance

### The −930A>G polymorphism and clinical phenotype

There was no correlation between genotype variants of the −930A>G polymorphism and MI or the severity of atherosclerosis estimated on the basis of the number of coronary stenoses or critical arterial occlusions observed during a coronary angiography (data not shown).

### Gene—traditional risk factors interactions

In the next step, we analyzed potential associations of respective genotypes and classical risk factors of CAD using the Pearson’s correlation model and we showed that the −930G allele carrier state was correlated with overweight/obesity (BMI ≥ 25) (*r* = 0.36, *P* = 0.04) and the AA genotype with cigarette smoking (*r* = 0.22, *P* = 0.03). Other tested parameters such as serum lipid levels, age, gender, a familial history of CAD, hypertension and diabetes mellitus did not correlate with genotypic variants of the *CYBA* gene polymorphism (data not shown).

Further statistical analyzes focused on interactions of the *CYBA* −930A>G polymorphism variants and the two classical risk factors of CAD. We found a synergistic effect of the −930G allele with overweight/obesity (BMI ≥ 25) and cigarette smoking (Table 3). Overweight/obese −930G carriers had an increased risk of CAD (OR 6.03, *P* < 10^−6^) compared with AA homozygous subjects with overweight/obesity (OR 2.50, *P* = 0.08) and −930G allele carriers with BMI < 25 (OR 1.44, *P* = 0.28). Estimated CAD risk was about 160 % greater than that predicted by assuming additivity of the effects (SI = 2.59). We also found that the −930G allele increased the risk of CAD associated with an exposure to cigarette smoking (Table 3). The observed effect was about 40 % greater than that predicted from the effects’ additivity (SI = 1.39).

**Table 3:** Synergistic effects between −930G allele carrier state (GG+AG genotypes), overweight/obesity (BMI ≥ 25) and cigarette smoking exposure

Genotype variant	Traditional risk factor	CAD (*n* = 240)	BD (*n* = 240)	OR (95 % CI), *P*	OR	SI
−930G (GG+AG)	BMI ≥ 25					
0	0	14	35	1	–	
0	1	11	11	2.50 (0.88–7.07), 0.08	OR_01 versus 00_	
1	0	80	138	1.44 (0.74–2.86), 0.28	OR_10 versus 00_	
1	1	135	56	6.03 (3.01–12.06), <10^−6^	OR_11 versus 00_	2.59
−930G (GG+AG)	Smoking					
0	0	8	32	1	–	
0	1	17	14	4.86 (1.70–13.87), 0.023	OR_01 versus 00_	
1	0	96	140	2.74 (1.21–6.21), 0.013	OR_10 versus 00_	
1	1	119	54	8.81 (3.81–20.39), <10^−10^	OR_11 versus 00_	1.39

*CAD* coronary artery disease patients, *BD* blood donors, *BMI* body mass index, *OR* odds ratio, *OR*_01 versus 00_ OR for traditional risk factor exposure, *OR*_10 versus 00_ OR for genetic risk factor exposure, *OR*_11 versus 00_ OR for co-exposure to genetic and traditional risk factor, *SI* synergy index

**Fig. 1:**
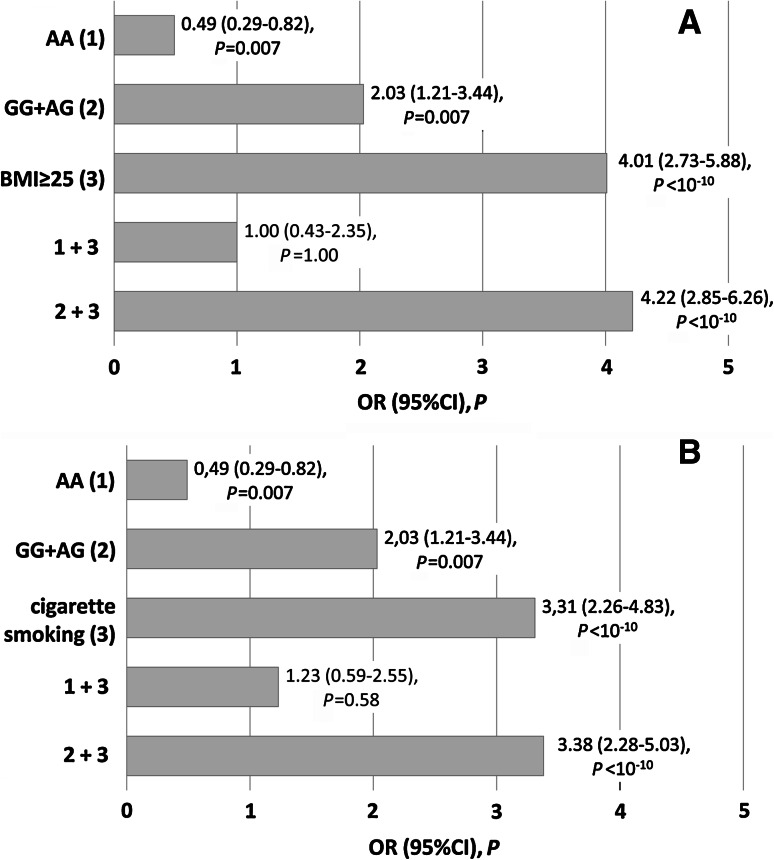
The influence of genotype variants of the *CYBA* gene −930A>G polymorphism on the risk of coronary artery disease associated with overweight/obesity (**a**) and cigarette smoking (**b**) in additive model of interaction

We confirmed the obtained results using an additive model of association. Overweight/obese −930G allele carriers (*n* = 135 in CAD group, *n* = 56 in BD group) were more frequent in the CAD group (56.3 vs. 23.3 %, *P* < 10^−10^). The power of this comparison was 99 with 99.9 CI. The frequency of overweight/obese −930A allele carriers (*n* = 11 in CAD group, *n* = 11 in BD group) did not differentiate groups of patients and blood donors (4.6 vs. 4.6 %, *P* = 1.00). Similar tendency was observed in the case of cigarette smoking. Smokers carrying the −930G allele (*n* = 119 in CAD group, *n* = 54 in BD group) were more frequent in the patients group (49.6 vs. 22.5 %, *P* < 10^−10^). The power of this test was 99 (99.9 CI). Figure 1 shows OR values obtained from these comparisons in a univariate analysis (Fig. 1). In the present study the overweight/obesity was a risk factor only in carriers of the −930G allele (Fig. 1a). Similarly, the risk of CAD associated with cigarette smoking was significantly lower in AA homozygotes than in −930G allele carriers (Fig. 1b). Both SI values as well as the results of additive association analyses indicate that overweight/obesity and cigarette smoking increase the risk of CAD especially in the −930G allele carriers.

Finally, we compared BMI values between AA homozygotes and −930G allele carriers in both patients and controls (Table 4), and we have shown that the BMI values differentiated only −930G allele carriers (*P* = 0.0004), while were almost identical in AA homozygotes from the groups of patients and controls (*P* = 0.98). BMI values of patients carrying the −930G allele correlated with triglycerides levels (*r* = 0.36, *P* = 0.002) and negatively correlated with HDL levels (*r* = −0.24, *P* = 0.015). Interestingly, these correlations were not observed in carriers of the −930G allele from the control group.

**Table 4:** Comparison of BMI values in carriers of AA genotype and −930G allele (GG and AG individuals) of the −930A>G *CYBA* gene polymorphism

GROUPS	BMI values (mean ± SD)	
	AA	GG+AG
CAD	26.38 ± 5.06	27.24 ± 4.10^a^
BD	26.37 ± 4.64	25.37 ± 3.05
CAD+BD	26.37 ± 4.79	26.55 ± 3.86

*CAD* coronary artery disease patients, *BD* blood donors

^a^CAD versus BD, *P* = 0.0004

## Discussion

In the present study we showed that the −930G allele carrier state of the *CYBA* −930A>G polymorphism is associated with premature coronary artery disease. To date, there was only one study analyzing the association of the −930A>G polymorphism with CAD conducted on the Austrian population [15]. Surprisingly, the authors found that the −930G allele was prevalent in the control group (*P* = 0.045). It seems that the observed contradictions resulted mainly from differences such as ethnicity and inclusion/exclusion criteria between Polish and Austrian populations. Both CAD patients as well as controls of the Austrian study were younger and not age matched (*P* < 0.001), in contrast to the Polish groups. The groups were not numerous (*n* = 100 for CAD patients, *n* = 200 for controls) and, it should also be added, that such a low frequency of −930G allele carriers (76.5 % in Austrian CAD group) was not observed in any of the major studies on the −930A>G polymorphism.

We did not find any associations of the analyzed polymorphism with hypertension. However, hypertension seems to be one of the main ways by which the −930G allele affects vascular functioning, according to the current state of knowledge [4–6, 10]. There are also other probable mechanisms of actions of the −930G allele, independent from hypertension like insulin resistance mediation and influence on the vascular wall structure, which will be discussed below.

It was shown that the −930G allele influences the expression of *CYBA*, especially in hypertensive subjects. *CYBA* mRNA levels were higher in GG than AA/AG hypertensives (*P* < 0.05). No differences in *CYBA* mRNA levels were found between genotypes of normotensive subjects [5]. Additionally, the superoxide production was increased only in hypertensive GG subjects but not in GG homozygous normotensives [5, 6]. Interestingly, a more recent study demonstrated that the −930A>G polymorphism may be a determinant of peripheral and central pressures also in normotensive individuals [16]. Finally, the −930A>G polymorphism was reported to be associated with hypertension in the Spanish population [4, 6] and Japanese males [10].

We also found a synergistic effect of the −930G allele with overweight/obesity (BMI ≥ 25) on CAD risk. The results of alternative, logistic regression model analysis showed that overweight/obesity is a risk factor for CAD only in carriers of the −930G allele. We also observed that BMI values differentiated only carriers of the −930G allele from the groups of patients and controls, while they were almost identical in AA homozygotes.

There are many interactions between NADPH oxidases and overweight/obesity. Fortuno and colleagues demonstrated that phagocytic NADPH oxidase activity was increased in obese subjects (*P* < 0.05) and was related to preclinical atherosclerosis in this condition [17]. Also animal studies have provided interesting results of diet-induced obesity on the NADPH oxidases activity and expression of its subunits. In the study conducted on obese rats, NOX4 expression was increased by three-fold in the aorta [18]. Additionally, upregulations of p22phox and p47phox in the adipose tissue as well as NOX4, p22phox, and p47phox in kidney were observed [18]. Very recent studies on mice provided evidence that dietary obesity increased both vascular [19] and hepatic [20] NADPH oxidase activity, which was associated with an enhanced expression of NOX2 [19, 20], p22phox and p47phox [20].

Another aspect of linking the NADPH oxidases with overweight/obesity is the impact of free radicals on insulin resistance condition. The study of Sukumar et al. [21] identified NOX2 as a central molecule in insulin resistance-mediated oxidative stress and vascular dysfunction. They demonstrated that higher levels of O_2_^−^ in insulin-resistant endothelial cells were inhibited by gp91ds-tat (NOX2 inhibitor). Double transgenic mice with endothelial-specific insulin resistance and deletion of NOX2 showed a reduced O_2_^−^ production and an improved vascular function [21]. These findings were complemented by results of another study [19]. Aortic vessels from obese mice after middle age had significant increases in NOX2 expression and ROS production, which were accompanied by a reduced insulin receptor expression [19]. Interestingly, there are presumptions that the −930G allele of the *CYBA* polymorphism is associated with insulin resistance in obese adult humans (*P* < 0.05) [22].

In the present work we also found that the risk of CAD associated with cigarette smoking was significantly higher in −930G allele carriers than in AA homozygotes.

Many studies demonstrated the role of NADPH oxidases in the promotion of cigarette smoking-dependent oxidative stress. NADPH oxidases were activated by many water-soluble components of cigarette smoke [23, 24]. Short exposure of human and animal endothelial cells to cigarette smoke extracts resulted in a large increase in O_2_^−^ production, which was inhibited by several NOX inhibitors [25]. An increased NADPH oxidase activity was observed among others in the ventricular remodeling induced by tobacco smoke exposure [26]. The −930A>G *CYBA* polymorphism was also analyzed in the context of an association between cigarette smoking and carotid intima-media thickness (IMT), a marker of subclinical atherosclerosis [27]. The −930G allele modified the strength of the association between cigarette smoking and IMT in young healthy adults. The differences in the mean and maximal IMT were most significant in subjects with the GG genotype, borderline significant for the GA genotype, and nonsignificant for the AA genotype. GG homozygotes had a higher mean and maximal IMT compared with the carriers of the A allele among smokers (*P* = 0.021, *P* = 0.012, respectively).

In our previous studies we showed associations of other *CYBA* gene polymorphisms with smoking, namely the 242C>T polymorphism [11] and the 640A>G polymorphism [28]. Carriers of 242T and 640G alleles were particularly at risk of the effects of exposure to tobacco smoke. It should be mentioned that there was no independent association of the analyzed polymorphisms with CAD, neither in these studies nor in the study on genetically related Slovenian population, where the C242T polymorphism was not associated with carotid atherosclerosis in patients with type two diabetes [29]. Although the results of the current meta-analysis have indicated that the 242T allele carriers have a marginal CAD risk increase (21 %) but only among Caucasians [30]. These two functional polymorphisms are not in linkage disequilibrium with −930A>G. It is however possible that the latter also affects the susceptibility to CAD, especially in smokers.

A limitation of the present study is the fact that the analyses were performed on multiple subgroups with a relatively small number of participants. Neither did we analyze the activity of NADPH oxidases nor the expression of p22phox. The relatively low frequency of hypertension in the control group (2.3 %) should also be discussed here as a potentially interfering factor. Such a low incidence may result from two main reasons. The first are the guidelines of the Polish Centres of Blood Donation and Blood Treatment, according to which the blood samples can be obtained only from patients with systolic BP less than 140 and diastolic BP less than 90 mmHg, on the day of blood collection. The second reason may be the recruitment criteria, which excluded donors with a familial history of CAD. This could have led to a further decrease in the observed incidence of hypertension. It is possible, however, that the frequency of hypertension is underestimated in this group, especially in the context of research conducted in a different group of Polish blood donors, where the frequency of hypertension exceeds 50 % [31]. In comparison, the frequency of hypertension in the general Polish population is in the range 30–45 % due to heterogeneous results of numerous epidemiological studies [32]. We do not think, however, that the declared low incidence of hypertension in the control group has influenced the outcome of the present study. Underestimating of hypertension frequency would affect the results of association studies on hypertension. However, we did not analyze such a relationship in our study, but the association of the −930A>G polymorphism and coronary artery disease.

In summary, we found that the −930A>G polymorphism is associated with coronary artery disease in the Polish population. The synergistic effects of this polymorphism with overweight/obesity and cigarette smoking increase the risk of CAD, and the −930G allele carriers are particularly at risk of consequences of obesity and tobacco smoke exposure.
